# It Starts with a Conversation: The Importance of Values as Building Blocks of Engagement Strategies in Community-Centered Public Health Research

**DOI:** 10.3390/ijerph18062940

**Published:** 2021-03-13

**Authors:** Ewelina M. Swierad, Terry T.-K. Huang

**Affiliations:** 1 Neurology Department, Columbia University Irving Medical Center, New York, NY 10033, USA; es2818@cumc.columbia.edu; 2 Center for Systems and Community Design, Graduate School of Public Health & Health Policy, City University of New York, New York, NY 10027, USA

**Keywords:** community-centered research, values, underserved communities, minorities, low-income, benevolence, universalism, self-direction, community engagement, community-based research, Value-Based Framework for Community-Centered Research

## Abstract

This study examined the life-motivating values of residents in underserved minority communities to inform the development of community engagement strategies. Semi-structured interviews were conducted to explore the main research questions: (1) what were the values of research participants, and (2) what did they consider important in their lives? The participants included twenty-seven ethnically diverse individuals living in low-income neighborhoods in New York City (NYC). Thematic analysis was performed to identify common themes and patterns related to the values that participants considered important in their lives. Three broad themes were identified: (1) benevolence; (2) universalism, and (3) self-direction. Benevolence implies a sense of belonging as the central meaning in life; community engagement strategies focused on this value emphasize concern for the welfare of loved ones. Community engagement strategies focused on universalism emphasize social justice and concern for the environment and the world. Finally, community engagement strategies focused on self-direction seek to satisfy participants’ needs for control, autonomy, and mastery. This study introduces the Value-Based Framework for Community-Centered Research. It illustrates how value exploration is central to a community-centered approach to public health research and can be an important first step for designing studies that are better aligned with community needs and contexts. Such an approach can also help to co-create a “research identity” with community members and integrate their values into a project’s purpose, thereby increasing community ownership and engagement in the study.

## 1. Introduction

Community engagement is a process that is increasingly seen as crucial in the development and implementation of effective health interventions designed to address the needs of culturally diverse communities [1,2]. Research suggests that values are building blocks of accountability, trust, reciprocity, respect, solidarity, and collaboration in community health research; researchers highlight the importance of understanding community values to better engage research participants and provide support and capacity-building for greater equity and justice [3]. To date, community engagement has mostly focused on community input into the development of public health interventions, with an eye toward tailoring messages and disseminating findings among stakeholders [4]. However, academic organizations face challenges in gaining insight into research participants’ deep-seated values to render community projects truly community-centered [5]. Indeed, effective and streamlined strategies, mechanisms, and specific steps to enhance this process remain unclear. The synergistic, iterative effects of value exploration on the trajectory of a given research project is also not yet sufficiently understood [5].

**Figure 1:**
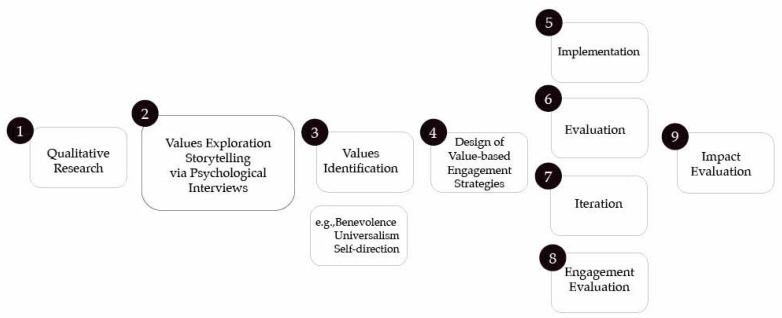
Value-Based Framework for Community-Centered Research.

In recent years, health researchers have started recognizing the necessity to develop effective community engagement strategies through both relationships with community members and knowledge and expertise outside of academia [6,7]. This approach is crucial for social change because it facilitates multidisciplinary knowledge co-creation to solve social problems that affect real people and their communities. [8,9]. Given that community research often concerns commitment to social justice and responsiveness to the values and needs of community members, we propose a humanistic approach that serves as the basis of a *Value-Based Framework for Community-Centered Research* (see Figure 1). Integrating the principles of participatory research [9], humanistic psychology focused on authenticity, compassionate social action, meaning [10,11,12], and human-centered design [13,14,15], this framework encapsulates the process of community-centered research. It highlights the importance of exploring participants’ values and life meaning as a first step to designing effective and culturally sound community engagement strategies in research.

Research suggests that values are critical because they inform and guide individuals’ behaviors [16]. Values provide compass in our lives, giving us direction and inspiring action [16]. In the context of our framework, through careful design of a qualitative study (Step 1), researchers explore participants’ stories via in-depth psychological interviews (Step 2) and identify participants’ core values (Step 3). Step 4 involves the design of the actual study and its value-based community engagement strategies followed by their implementation (Step 5), evaluation of the initial community engagement strategies (Step 6), iteration of the original strategies (Step 7), evaluation of refined participants’ engagement (Step 8), and evaluation of the long-term impact on research process and outcomes (Step 9). The *Value-Based Framework for Community-Centered Research* aims to incorporate participants’ core values in every stage of the research project: its design, implementation, and evaluation.

Successful community engagement that boosts trust in health research involves several qualities that researchers need to demonstrate, such as loyalty, respect, honesty, competence, and shared-value creation [3,17,18]. Community engagement is often concerned with creating shared value among multiple stakeholders [4]; hence, it is critical that impactful community-centered research with underrepresented groups reflect the voices and values of these communities. Participants need to trust researchers, develop an identification with a research project, and be valued and protected [4] as according to research, one of the essential ingredients of a meaningful community engagement process must include trust between researchers and all stakeholders [19]. Trust depends on the quality of interactions between researchers and community members (research participants) [20]. However, building and maintaining trust with underserved communities have proven to be difficult [21]. Some of the reasons for the mistrust in community research among disadvantaged groups involve mistreatment and stigmatization of these groups [22,23], the historical abuses such as those associated with the Tuskegee Study, and concerns of ethical misconduct [24]. In addition, lessons learned from environmental research demonstrate that the ways in which communities are engaged is one of the critical factors likely to determine whether community engagement is successful [25]. Evidence from environmental research indicates that there are often differences between community and government perspectives on what constitutes ‘successful’ community engagement. Government perspectives highlight transparency while aligning to government policies and priorities, whereas community perspectives emphasize independence, respecting social and geographical context and avoiding burnout. For the community engagement to be worthwhile, it has to provide value to community members and be genuine [26]. Taken together, to optimize participants’ recruitment, engagement, and retention, it has been suggested that community engagement should focuses on the following: (1) deep understanding of individuals’ needs and values; (2) cooperative development of all steps involved in the process of research/infrastructure development; (3) co-learning for mutual benefits of all parties involved in the research process; and (4) using data to inform and engage participating individuals [27,28].

The current paper addresses the first four steps of the proposed *Value-Based Framework for Community-Centered Research* in depth—i.e., qualitative research, values exploration, values identification, and design of value-based engagement strategies. These first four steps provide an opportunity to honor, focus, and reflect on the experiences of those most directly affected by issues in our communities enhancing connection and trust between community members and all the stakeholders involved in a given community research project [3]. Although steps 5 to 9 (implementation, evaluation, and impact factors related to community engagement strategies) are beyond the scope of this manuscript, they constitute integral next steps of value-based community-centered research; thus, they are shown as part of the overarching framework in Figure 1. Through in-depth interviews and empathic listening, the current study aimed to uncover research participants’ values that could inform the development of community engagement strategies embodying these values.

## 2. Materials and Methods

### 2.1. Research Design

Participants were asked multiple semi-structured and open-ended questions that explored the values, worldviews, and beliefs that guided their behaviors. These questions were developed based on the values theory [29], Maslow’s hierarchy of needs [30], identity signaling [31] and identity-based motivation [32]. The format and implementation of the interviews were inspired by acceptance and commitment therapy [33] that offers a safe and nonjudgmental context focusing on acceptance, presence, and values-based living. Additionally, the lead author of this paper, who is a psychologist and conducted the interviews, used validation, unconditional positive regard, and concreteness to ensure active listening and create a supportive environment [34]. Such context-setting facilitated an exploration of questions focused on personal stories and experiences. This study was approved by the Institutional Review Board of the City University of New York.

### 2.2. Participants and Recruitment Strategies

Participants included twenty-seven ethnically diverse individuals living in low-income neighborhoods in New York City (NYC). They were selected from the Physical Activity and Redesigned Community Spaces (PARCS) Study, an ongoing longitudinal study that aims to examine the impact of citywide park redesign and renovation on health and wellbeing [35]. Participants were randomly selected by another researcher not involved in the current qualitative study. The lead author of this paper, who is otherwise not involved in the recruitment or data collection of the larger PARCS study, then contacted individuals on the list by phone to invite them to participate in the interview.

The PARCS Study included adults ≥18 years of age with no mobility issues and who understood/spoke English, Spanish, or Chinese (Mandarin or Cantonese) across 54 low-income neighborhoods throughout New York City. Out of the 27 participants of the current qualitative study, 22 were women, 5 were men (gender representation was reflective of the PARCS Study participants’ gender distribution in part because of an oversampling of mothers in a subsample of parent–child dyads); 6 self-identified as Hispanic or Latino/a, seven as Black or African American, six as White or Caucasian, four as Other (the remaining four did not indicate their ethnicity). Nine participants were severely low income, with an annual household income of less than USD 20,000, while the remaining participants reported annual household income of USD 20,000 or above or more. The participants represented diverse age groups; two were between 20 and 30 years old, ten were between 30 and40 years old, five were between 40 and 50 years old, eight were between 50 and 60 years old, and two were between 60 and 70 years old. The majority of participants were either married/partnered (*n* = 11) or never married (*n* = 9). The remaining participants were divorced (*n* = 2) or widowed (*n* = 2); three participants did not disclose marital status.

### 2.3. Procedure

Commencing each brief interview, the interviewer introduced herself and explained the purpose of the study. After providing informed consent, the interviewer read the interview questions in sequence (Appendix A) and engaged in clarification, follow-up or probing questions when needed. Interviews were held over seven months, and the interviewer met with the participants at their local cafés (Step 1 and 2). The 45-minute-long interviews aimed to explore the main research questions: (1) what were the values of research participants? (2) What did they consider important in their lives? Questions were pilot-tested with three independent participants to ensure their cultural appropriateness. The interview format enabled us to obtain detailed insight into participants’ values.

### 2.4. Thematic Analysis

The interviews were transcribed and de-identified. Each participant was assigned a number (e.g., “*P1*”). Interview transcripts were then imported into NVivo v.10 (QSR International, Burlington, MA, USA), a qualitative data analysis software package. Thematic analysis [36] was used to identify common themes and patterns related to the values that participants considered important in their lives.

The specific qualitative method used in this study recognizes language as a tool for storytelling that reflects people’s broader social and physical environments (Steps 1 and 2) [36]. In other words, thematic analysis highlights how people make sense of their experiences that are informed by their social and environmental context [36]. Understanding the meaning of people’s experiences seems to be particularly important in the case of members of disadvantaged groups (who represent the majority of PARCS study participants) that are often discriminated against and exposed to prejudice and social injustice [37].

The interview data were analyzed following the thematic analysis guidelines created by Braun and Clarke [36] (Step 3). During each stage of the analysis, the authors of this manuscript discussed the emerging themes to ensure consistency within the patterns identified. These findings informed potential value-based strategies for community engagement as part of Step 4 of the proposed framework.

## 3. Results

Table 1 presents three broad themes that were identified throughout the interviews based on Step 3 of our *Value-Based Framework for Community-Centered Research* framework. All themes were related to values that participants described as the most essential in their lives: (1) Benevolence, (2) Universalism, and (3) Self-direction. These values were critical for participants in the context of engaging in research; thus, identifying and exploring these values represented the first step in community-centered research. Based on these values, researchers could design value-based engagement strategies to optimize alignment with participants’ core values. For Benevolence, community engagement strategies should emphasize concern for the welfare of closest family members, loved ones, and friends (e.g., sense of belonging, meaning in life, a spiritual life). For Universalism, these community engagement strategies should highlight a concern for the welfare of those in the larger society and world and nature (e.g., social justice, the world of beauty, protecting the environment). Finally, in the context of Self-Direction, these community engagement strategies should give people opportunities to satisfy their needs for control, autonomy, and mastery (e.g., creativity, freedom, privacy).

There is considerable interest in community value exploration to enhance community research and participant satisfaction [3]. Our findings can be examined in the context of decades of psychological research that suggests that human values are the foundations of individual actions and thoughts, and when a program or service matches people’s values, they are more likely to evaluate it positively and to engage with it more frequently [29,38]. In the same vein, we discuss our findings below in the context of psychological theoretical concepts of values identified in the literature [39]. Schwartz [29] distinguishes ten fundamental values that reflect a specific motivational goal. These values (and goals) include: (1) Self Direction (independent thought and action, choice, creativity, exploration); (2) Stimulation (excitement, novelty, and challenge in life); (3) Hedonism (pleasure and sensuous gratification for oneself); (4) Achievement (personal success through demonstrating competence according to social standards); (5) Power (social status and prestige, control or dominance over people and resources); (6) Security (safety, harmony, and stability of society, of relationships, and of self); (7) Conformity (restraint of actions, inclinations, and impulses likely to upset or harm others and violate social expectations or norms); (8) Tradition (respect, commitment, and acceptance of the customs and ideas that traditional culture or religion provide the self); (9) Benevolence (preserving and enhancing the welfare of those with whom one is in frequent personal contact (the ‘in-group’); and (10) Universalism (understanding, appreciation, tolerance, and protection for the welfare of all people and for nature). As presented below, we have identified three of these values in our qualitative work and we analyzed them through the lens of human-centered research, with practical implications for community engagement.

### 3.1. Theme 1: Benevolence

This theme reflected the value of benevolence, as expressed in participants’ love and care for the well-being and safety of their loved ones and immediate social circles (so-called in-groups). The majority of participants mentioned that family and friends were the most critical motivational and supportive forces in their lives. This theme revealed participants’ dedication to well-being of in-group members and the importance of caring, dependability, and trustworthiness in close relationships. Three sub-themes were identified: (1) family and friends as a source of emotional support, encouragement, and inspiration; (2) intergenerational learning and learning from each other to help one another; and (3) a Golden Rule of treating others the way you want to be treated.

#### 3.1.1. Benevolence: Family and Friends as Sources of Emotional Support, Encouragement, and Inspiration

The majority of participants perceived their family and friends in the context of social support that they gave and received from their loved ones, sharing, and a way to enjoy their time together. Sometimes participants discussed social support as a way of providing better opportunities for one’s family. Friends and family were perceived as “the calming medicine” and inspiration for life:

**P12:**  *“My family inspires me—I want them to be healthy, happy, and comfortable (…), making sure my family is safe is important to me. My mother is one of the biggest role models in my life—she has accomplished a lot, overcoming a lot of obstacles; she kept pushing forward in the face of challenges (…) When I experience challenges, I look for my family for support.”*

**P10:**  *“Being an immigrant is sometimes hard, but I want to make a better life for my son (…) it is important to me to provide my son with nice and calm environment so that he can enjoy his childhood—I’m trying to create good childhood memories for him, so when he grows up he remembers all the nice experiences.”*

**P1:**  *“Family and friends are the most important to me—even now that some of my family is in Israel, it is so easy to stay in touch. Before, years ago, it was so difficult to call them. I had to buy phone cards. Now it is so easy and free, and I can see them too, see their pictures—I’m in touch daily with my family (…) My family keeps me going; they are the reason for which I do what I do, the reason for waking up every morning. When I have a bad day when I suffer from migraines, my kids keep me going—my sons are definitely momma’s boys.”*

#### 3.1.2. Benevolence: Intergenerational Learning and Learning from Each Other to Help One Another

According to participants, benevolence often entailed intergenerational learning. Many participants discussed their love for their children and loved ones (who as **P16** noted “always come first”) in the context of intergenerational learning. Very often, the intergenerational lessons revolved around values, ethics, and family moral code, as well as a sort of torch burning brightly before handing it onto the next generations. Intergenerational learning often seemed to be bi-directional: older members of a family taught younger members, and younger members taught parents and grandparents. Additionally, sometimes, what one learned from the older generation (e.g., their own parents), one paid forward:

**P4:**  *“I teach my children to keep in contact with their cousins and their grandparents on both sides—it makes a difference because if you teach kids to love one another, you teach them to love everybody, there is no chance for negativity. I think racism is taught, it is not something you are born with, so when you teach your kids to love, you teach them to love everybody, independent on others’ skin color or ethnicity.”*

**P10:**  *“My son and I learn from each other (…). We teach each other—I have never been a mother before, so he teaches me how to be a better mother. I did not have an easy childhood, had nobody who cared to listen to me, so I’m trying to do the opposite of what I have learned and experienced as a child with my son to make sure he is happy. As a parent, you are just a parent, not a general in the military to give your kid orders—it is a two-way street, you learn from each other.”*

#### 3.1.3. Benevolence: The Golden Rule of Treating Others the Way You Want to Be Treated

Participants discussed the value of Benevolence in the context of care and respect for the well-being of other people in one’s community with whom they interacted daily, not only one’s family and friends. Many participants expressed the idea of Benevolence as it related to karma and the “Golden Rule:” Treat others how you want to be treated. In the context of the “Golden Rule,” generosity (giving attitude) was a common sentiment among many participants:

**P4:**  *“I always say, ‘you treat people the way you want to be treated, you want respect, you have got to give respect’ (…) I always teach my kids, ‘the world does not owe you anything, so if someone gives you something, say, ‘thank you.”*

**P1:**  *“One of my mottos is ‘just give to give, don’t give to receive,’ my mother is more of ‘give to receive’ type of person (…) I’m just like my father. I give to give (…) I like having people over, it fills up my house, I cook, and I do parties for them, it is worth it—I believe in karma, what goes around, comes around.”*

#### 3.1.4. Benevolence: Practical Implications Regarding Value-Based Engagement Strategies for Community-Centered Research

In Step 4 of our Value-Based Framework for Community-Centered Research, we propose that researchers design value-based engagement strategies to optimize the alignment with study participants’ values. In order to increase participants’ engagement in a community-centered research, research may consider the following strategies focused on benevolence (this list of strategies is meant to inspire rather than be used as a fixed recipe): (1) designing materials used for the study (e.g., cards, posters, surveys) that communicate clearly the purpose behind and core values of the project. This strategy builds loyalty to and identification with the project. These materials also incorporate (whenever applicable) quotes from participants that focus on benevolence; (2) selecting incentives that are aligned with participants’ core values, such as benevolence, and that include a message embodying participants’ values (e.g., water bottles with the project’s logo and meaningful quotes from the interviews); (3) sending participants handwritten Thank You notes as well as birthday cards to express genuine gratitude and respect for their participation; (4) designing infographics that simplify all the steps involved in the study (e.g., a calendar for study activities to reduce the burden of participation and to communicate with transparency and clarity; fridge magnets that include necessary information, etc.); (5) emphasizing the idea of sharing what study participants learn during their participation in the study with family and loved ones; (6) exhibiting respect, transparency, clarity, and kindness through every mode of communication with participants; (7) designing clear study materials to respect participants’ time needed to involve in the study. These strategies can help with building a sense of community among study participants to increase their identification with and attachment to the project. These strategies can be implemented through creative use of art and design, as well as in-person and other forms of communication.

### 3.2. Theme 2: Universalism

In addition to the value of benevolence and its emphasis on love and care for one’s family, friends, and communities, many participants considered universalism a critical personal value. It signifies a higher purpose in life that ensures the welfare of other people, animals, and the environment. Universalism revolves around understanding, appreciation, tolerance, and protection for all people’s welfare and nature. The theme incorporates three sub-themes: (1) nature and a clean environment; (2) making a positive contribution in the world; and (3) societal concern and appreciation of diversity (e.g., equality, social justice) and the complexity of human nature.

#### 3.2.1. Universalism: Nature and a Clean Environment

Many participants discussed the importance of nature and a clean environment in their lives. They expressed their commitment to protecting and preserving the beauty of nature for future generations. Many participants noted that happiness could be a direct outcome of a clean environment:

**P13:**  *“Parks mean life to me (…). I think the community is something we should all care about because we are the community, and we have to take care of our community, enjoy it, learn from it (…) I want my kids to be part of something meaningful and purposeful—and that is why we participate in the PARCS project.”*

**P1:**  *“What is important to me now is happiness and health, and it often has to do with the environment, it is about leaving this place better and greener for the next generation (...) Health is essential in life, and you have to have good schools, housing, and a clean environment to get it. You cannot have good health if you do not have a good environment.”*

#### 3.2.2. Universalism: Making a Positive Contribution in the World

Participants expressed their belief that making a positive change in the world, however small, was essential for them, even if one did not get instant recognition for it. Participants discussed how they engaged in volunteering and giving back to their community by using their skills and talents to make a difference in their communities. As participants noted, helping others aimed at both “doing things for other people” and “staying positive,” so volunteering could be personally gratifying as well. Sometimes giving back was inspired by having been a beneficiary of someone’s kindness in the past.

**P26:**  *“Do good anyway, even though nobody appreciates you, nobody sees you. Do good anyway, just for your own, for others, and for the world, even if they don’t reward you. Do good anyway. Even though you feel you may fail, do good anyway. Just do your best, whatever you can, don’t expect perfect, mistakes are just stepping stones to success, just continue on. Maybe through mistakes, you actually change the path to a better path; show appreciation to others if you can because other people like to be appreciated.”*

**P5:**  “*I volunteer for an animal shelter, too. I have always loved working with animals, they live in a completely different world that is also intertwined with ours, but you can’t know what they are thinking or feeling, so it is just fascinating to observe (…) Thinking about the world in general, animals, nature, people help me keeps things in perspective and not to take myself too seriously; it keeps me grounded.”*

#### 3.2.3. Universalism: Societal Concern, Appreciation of Diversity (e.g., Equality, Social Justice), and the Complexity of Human Nature

The value of universalism often embodied participants’ care about social justice. Many participants discussed the importance of universalism, social justice, and activism in the context of their race and culture. Some participants talked about the critical role of our history in shaping their appreciation of social justice. Participants also discussed how we should celebrate our similarities and differences rather than focusing on what divided us:

**P6:**  *“Activism, social justice issues are very important to me (...) I believe that minority groups slowly energize and mobilize themselves to bring about change. Still, this change happens slowly, just like Martin Luther King said, ‘the wheels of justice turn slowly,’ but they make a difference (…). I wish I were more of an activist like Malcolm X who fought for social equality, read a lot, did his homework to be prepared to engage in all sorts of ideas and beliefs, to discuss them, to confront them. I believe that we all need inspiration when it comes to justice, we need leaders because not everybody has that special light like Jimmy Hendrix, to keep going, to keep writing songs, to keep on pushing (…) Why only some people have that?? Hendrix, Malcolm X, Martin Luther King, Bob Marley, Muhammad Ali, Obama, Steve Wonder—these people are my true inspiration. I gravitate towards people who made a difference coming from the bottom—that makes me say, ‘wow. I could never do what these people did (…) When Obama became a president, it was a pivotal moment for me—I recall watching TV with my small daughter who—when she saw Obama, and I asked her who it was, she said ‘papa’. I said to myself that it was the first time in American history that your daughter could take you for a president (…) it was a big thing to me, it made me feel like things are changing (…) it was a very powerful moment.”*

**P16:**  *“What fascinates me is that when I learn about other people’s cultures. I realize that we are not that different. Our foundation is always the same thing. We are more alike than we are different (…) I often wonder about what happens when, after we die, one person goes to heaven, and one person goes to hell. I often reflect on what happened that got them to this place; for example, kids who grew up on the streets can end up being thefts because they do not know any better.”*

#### 3.2.4. Universalism: Practical Implications Regarding Value-Based Engagement Strategies for Community-Centered Research

The universalism value can be applied in community-centered research to increase participants’ engagement through the following strategies: (1) organizing design thinking workshops with participants to identify innovative ideas for community-centered research (https://www.cunycscd.org/reports; accessed 15 Jan uary 2021); (2) joining local events to engage participants in a conversation about their communities and what is important to them, offering them food and small incentives. This strategy promotes shared value among study participants and enhances their engagement; (3) having the project’s website or social media channels communicate *universalism* as a core guiding value of the study. Each survey includes a brief comment, thanking participants for “sharing their voice with us, and, by doing so, making a difference in their communities and helping to shape the future for the next generation”; (4) recognizing community champions (with certificates) who are exceptional participants by highlighting their universalism value (e.g., their commitment to making our communities healthier and greener). These champions can be publicly recognized during events; (5) partnering with local charities or community-based organizations and sharing the list of these organizations with the participants so that they can choose to engage in volunteer work; (6) inviting participants to roundtables to talk about their communities and causes they are passionate about. Each roundtable can be organized around a specific theme (e.g., animal welfare, social justice, environment). The outcomes of these discussions (whenever applicable and appropriate) can be shared with the entire community. These strategies can be implemented through leveraging local assets, storytelling, web-based -engagement, roundtables, recognition of champions, etc. The goals of applying the universalism value to community-centered research is to communicate in a way that conveys a higher purpose while creating an opportunity for participants to express and engage in the activities of their choice and to promote the welfare of the broader society and of animals and the environment.

### 3.3. Theme 3: Self-Direction

The final theme was participants’ value of self-direction that embodied their desire to learn, grow, educate themselves, and pursue freely many different passions and interests in their lives. Many people expressed their gratitude toward any opportunity they got in life to learn and to become better versions of themselves. They appeared to perceive learning as something that, as **P16** noted: “*changes you, changes the way you look at life*.” As she later stated: “*The more you learn, the more you know about yourself.*” The self-direction theme incorporates three sub-themes: (1) self-directed learning as self-improvement, and problem-solving tool; (2) learning as a self-discovery: creating one’s own unique path; (3) self-direction as a pursuit of one’s hobbies and passions; (4) finding one’s tribe and seeking inspiration

#### 3.3.1. Self-Direction: Self-Directed Learning as Self-Improvement and Problem-Solving Tool

Many participants reflected on the concept of continuous learning and education as tools for practical problem-solving and a vehicle for a better future for themselves and their families. They saw learning as an opportunity for defining oneself to fully own one’s choices and decisions. Participants discussed the importance of recognizing that we must learn from the mistakes that we inevitably make and incorporate these learnings to move forward. Finally, participants often shared examples of mastering skills that directly improved their lives:

**P6:**  *“I think one of the best advices I got is Malcolm’s words: ‘Think for yourself’—you can get a million pointers from people, but you have to do what is right for you, what feels right inside. I try to incorporate that into my life. Don’t let people tell you, ‘you should not do this, or you should not do that’ (..) I met Bob Dylan, whom Hendrix really liked, he wrote: ‘Like a Rolling Stones’ song. It is about being who you are and making something out of yourself. ‘How does it feel to be on your own…’ Jimmy Hendrix ‘lived’ this song. What is it like to go out and make something out of yourself when people tell you are not good enough, you are not going to make it, you have no food? And you do it despite all of it. This song was perfect for Hendrix.”*

**P13:**  *“Live life every day. Embrace life. Have adventure. Go out and do things. Learn. Sometimes take on challenges. You may learn something new from it. Take on little challenges, take on obstacles. It does not matter if you always win; just don’t be afraid to try (…) When something that you do affects you negatively, or hurt you, just move on. But you should always try.”*

**P21:**  *“I read a lot, and lately, I learned a lot about time management that really got me. Everyone has the same time; it all depends on how we manage it. Every time that you have devoted to the family make sure it is 100%. You are not thinking about work; this is your family time. When you are at work, it is your work time, so don’t stress about other things. So, this is it. What I really want to implement in my life is giving your full time and attention to whatever you are doing.”*

#### 3.3.2. Self-Direction: Learning as Self-Discovery: Creating One’s Unique Path

The majority of participants talked about the fact that there was always something to learn, and that being curious was what made life satisfying and fulfilling. In the spirit of self-direction, many participants reflected on their sentiment towards traveling and learning new cultures in order to learn about the world. They also discussed how self-directed learning helped them create and leave their legacy. In their pursuit of a unique life path, participants discussed the importance of trusting one’s intuition in this journey:

**P1:**  *“It is important for me to feel fulfilled, to grow continually, to learn (…) I love New York, I love Manhattan: when I first time visited Manhattan, I felt like I’m in a movie, it felt mesmerizing. Some people don’t like the city, and I’m like ‘how you don’t like it?’ It is so beautiful; it has so much energy. The people, the vibe, even the way people dress; it is different. You can be who you want to be, sit in a restaurant alone. I have never done that before coming to New York. You can go for coffee by yourself, go to the movies by yourself. People do that here all the time. I still do that (…) You can go to shows, museums; the city has so much to offer—you can learn so much.”*

**P14:**  *“It is not what you do, but how you do it. That’s my life philosophy; your actions speak a lot because I can be upset at something but take a few breaths and handle a situation well (…) My happiness comes from my achievements. I want to make sure that people remember me for something, like when you are a teacher, and my kids remember me for things. That makes me happy because kids are genuine. If they don’t like you, they will tell you—that’s what I always liked about working with children. They don’t know prejudice; they are taught that; they don’t know how to lie; they are taught to do that. I believe that people are genuinely good; it is what they learn that turns them into bad people.”*

#### 3.3.3. Self-Direction: Self-Direction as a Pursuit of One’s Hobbies and Passions

Participants often discussed self-direction in the context of their pursuit of hobbies and passions that help them live more meaningful and fulfilling lives:

**P20:**  *“I love taking pictures. I use my phone for that; I take photos of everything: the birds, the sky, the moon, the trees (…) It makes me thankful to be alive. My favorite ones are the photos of my kids...and the moon.”*

**P27:**  *“I like playing and coaching football—I think football is a way to instill some of the values like perseverance, hard work, doing your best every second of life—I’m always passionate of football, it is a metaphor of life.”*

#### 3.3.4. Self-Direction: Self-Direction in Terms of Finding One’s Tribe and Seeking Inspiration

Many participants shared stories about significant individuals, whether from pop-culture, history, or the past, who inspired their learning and growth. Those inspiring figures encouraged participants to pursue their passions and helped them deal with life challenges. Sometimes this type of self-directed inspiration came from music or reading:

**P6:**  *“One of my biggest inspirations is Jimmy Hendrix. I connect to his life journey. When I play guitar, I let the bit and the energy drive, fire my feelings. When I play, I connect with Jimmy, I’m there (…) if it weren’t for him, I would not sound like this—I learned the energy, and I connect with people when I play. I’m inspired by Jimmy’s words: “I want my music to go to the soul of the people, not just ears; to move you.”*

**P15:**  *“I look forward to reading a book called ‘Secret’—it is an inspirational book. What I want to get from this book is I want to see whether I can make things better for myself. After reading this book, one of my friends started traveling. And I want that for myself too. I want to be someone like her, open for experience. I want to do something different because I’m tired of doing the same things over and over again.”*

#### 3.3.5. Self-Direction. Practical Implications: Regarding Value-Based Engagement Strategies for Community-Centered Research

We propose increasing participants’ engagement in community-centered research through the value of self- direction by these strategies: (1) giving back to participants by sharing with them curated vides of a compilation of inspiring take-home messages from the interviews that they can reflect on and learn from (e.g., cards, calendars, notebooks, etc.); (2) inviting participants to submit monthly artwork—a song, poem, photograph, design work, etc. The research team can share the submission with others, showcasing participants’ talents and skills, and providing a platform for creative self-expression. The artwork can be shared with the participants via social media channels, email and/or text; (3) designing a monthly newsletter (e.g., a blog post) that is short, clear, and well-designed, and includes tips on the issues that participants care and want to learn about (e.g., kindness https://parcs.commons.gc.cuny.edu/2018/04/16/do-good-anyway-about-kindness; accessed 15 January 2021); (4) organizing mini-lectures (e.g., podcasts) on topics that participants care about (e.g., problem-solving, creativity); (5) providing participants with a list of available free resources in their communities that they can use to learn new skills; (6) partnering with socially conscious businesses in the community to provide samples of or discount on their products/services so that participants can learn about new products and explore new services (e.g., dance or parenting class); (7) organizing book/magazine swap events where participants can exchange items. In the spirit of self-direction, all these strategies aim at providing learning or growth opportunities for participants in areas that are not directly related to the study variables and outcomes.

### 3.4. Implications of Participants’ Values for Community Engagement Strategies

As mentioned above, in Step 4 of our Value-Based Framework for Community-Centered Research, value-based engagement strategies were designed to optimize the alignment with study participants’ values. Some of these strategies have been implemented by our research team in the context of the PARCS Study (Step 5); others are being considered in the future (Step 5). These strategies aim to achieve the following value-based goals: (1) Building a sense of community among study participants to increase their identification with and attachment to the project; (2) Communicating in a way that conveys a higher purpose while creating a chance for participants to express and engage in the activities of their choice to promote the welfare of the broader society and of animals and the environment; (3) Providing learning or growth opportunities for participants in areas that are not directly related to the study variables and outcomes. Some strategies are more cost-effective than others, and they constitute different options of a menu (there is not necessarily one strategy that fits all research types or study participants). Future research is warranted to focus on the next steps of the *Value-Based Framework for Community-Centered Research*, exploring the implementation and evaluation of these strategies in the context of longitudinal or intervention studies in the community (Steps 5–9) and examining their role in improving the research process (e.g., participant recruitment and retention) and study outcomes.

## 4. Discussion

Building on prior psychological research, this paper explored the fundamental values of participants in a large, ongoing community research study to inform strategies for meaningful community engagement. We propose in this paper *a Value-Based Framework for Community-Centered Research* that outlines a stepwise process for designing and implementing impactful and culturally sound research in the community. The current paper addressed the first four steps directly (i.e., qualitative research, values exploration, values identification, and design of value-based engagement strategies). The results of this exploratory study illustrate the three essential values that participants identified in their lives: benevolence, universalism, and self-direction. These, in turn, served as the basis for ideating community engagement strategies that echo participants’ values, identities, life experiences, needs, desires, and aspirations.

The study built on previous research that explored engagement strategies employed in community-based health research [5,40] but is distinct in its focus on values as a driving force of community engagement as opposed to other factors such as community outreach, collaboration or partnerships. Through in-depth interviews and empathic listening, we sought to discover participants’ personal life stories to allow their deeply held values to emerge (Steps 1 and 2). This methodology enabled researchers to develop trust, a critical ingredient for ethical and culturally sound research [41]. Following review of the themes identified from the interviews (Step 3), we translated the results into potential strategies for community engagement in health research (Step 4). We ideated community engagement strategies that echoed participants’ values, identities, life experiences, needs, desires, and aspirations. Through this study, we found that exploring values can be a powerful tool for public health researchers and practitioners to engage community members in solving societal problems. Values are incredibly important to people, connecting them to who they are and what they believe in.

The values of benevolence, universalism, and self-direction found in our study are corroborated by previous research suggesting that community-centered projects may need to (1) build authentic and trustworthy connection with people focusing on their wellbeing; (2) stand for a higher purpose and exhibit its core values and beliefs throughout any interactions with people; and (3) help people learn, grow, and solve their problems [40]. By building trust with research participants, researchers attend to participants’ value of benevolence. In the context of self-direction, participants themselves decide whether they want to be part of a study (self-direction). Once they are in a study, they can develop a strong relationship with the project based on their favorable experiences with the research team and other stakeholders involved in the project (benevolence). If the study focuses on the welfare of others, or other relevant social and environmental causes (such as PARCS), the loyalty to the project can be further enhanced through universalism. Developing a robust community-centered project identity can meet individuals’ value of universalism—a genuinely held value of something bigger than participants themselves, such as the improving or protecting the environment. Finally, through identification with core principles of a community-centered project, participants can express who they are to others (e.g., “I’m someone who care about the environment”) while having a chance to learn, develop new experiences, and explore new goals related to the project (self-direction).

One of the reasons why values are important in community engagement is that they drive individuals’ thoughts, feelings, and behaviors, and are connected to people’s essential identities [29,32]. Our findings are congruent with Maslow’s Pyramid of Needs, and his emphasis on self-actualization as one of the most important drivers of human behavior [30]. The findings also reflect the Identity-Based Motivation theory [32]. Based on this theory, individuals tend to engage in behaviors that reflect their important social identities [32]. Similarly, consistent with the Identity Signaling framework [31], if a research project conveys a particular symbolic “label” or identity, participants may adopt that label and become more engaged in the study. For example, just as in the case of the PARCS Study, if a study’s purpose is to improve the quality of urban space by creating a healthier environment for people, the study participants can identify themselves as the ones “who care about the environment.” This identification can be further reinforced by the self-image concept that suggests people are motivated to maintain a positive image of themselves.

The importance of values and their influence on people’s actions identified in this research is consistent with several other psychological concepts. For example, the Values Theory conceptualizes values as desirable goals, varying in importance that determines what is essential in people’s lives [29], influencing their emotions, motivations, and actions [42]. In addition, according to the Expectancy-Value Theory, people engage in the pursuit of what they value, including what they perceive as their basic needs, aligned with their beliefs, experiences, and a vision of who they may become as a result of this pursuit [43]. Similarly, Stern’s Value-Belief Norm Theory postulates that people follow personal norms and engage in constructive behaviors when they believe that violating the norms would adversely affect what they value [44].

In a community-centered approach to public health research, the benefits of engagement can be reciprocal. Researchers can learn about what matters in participants’ lives to improve the “fit” and rigor of the study in alignment with unique community needs and context, while participants can gain opportunities for learning and growth through their active engagement in research. As the goal is to co-create and co-own the ensuing research, this approach could help with addressing the issues of power differentiation in research. Research quite often involves the power of experts “imposing” their solutions on community members. Therefore, in addition to building values exploration into public health research, incorporating engagement strategies throughout a study, such as providing opportunities for learning, could significantly reduce the power disadvantage among participants [45].

In the exploration of participants’ values, it is essential to consider the process of psychological interviews. As some of the questions are personal, a researcher needs to create a supportive environment validating the potential fear of being exposed or feeling vulnerable. In the spirit of humanistic psychology, participants need to feel heard and respected; thus, empathic listening that provides positive emotional reinforcement encourages participants to share and ultimately creates an authentic connection [46]. Once a researcher earns participants’ trust, they often express gratitude for being able to share and explore their stories with the researcher. Sometimes people release “the burden of untold stories” that they carry with them, revealing their identities and values in the process. Embodying a humanistic approach to interviews, one of the most prolific interviewers of our times, Oprah, once said: “I’ve talked to nearly 30,000 people on this show, and all 30,000 had one thing in common: They all wanted validation (…) Can you see me? Can you hear me? Does anything I say mean anything to you?” Similarly, every psychological interview based on values exploration needs to reflect this simple principle. That is the foundation of trust and human connection.

Although the current study revealed valuable insights regarding individuals’ values and their implications for the design and implementation of community engagement strategies, some limitations should be acknowledged. First, our sample was focused on residents in low-income neighborhoods in New York City. Therefore, study findings may not be generalizable to all populations. Future studies can build on the insight derived from this research and examine different dimensions of values with a more extensive and more diverse sample of participants. Second, the degree to which participants adhere to the values identified in the study, as well as the rank of these values, may vary among participants, and qualitative methods cannot fully capture these variations. Third, the sample consisted predominantly of women—out of twenty-seven participants, only five were men (gender distribution was reflective of the PARCS Study). This might have influenced the results of the study. Future studies could further explore gender differences. Finally, based on the values identified in the studies, the engagement strategies proposed are exploratory and need further testing and evaluation. A future follow-up study could investigate how participants perceive and evaluate these strategies in the context of their values and the impact on study engagement and retention (Steps 5—9 of the *Value-Based Framework for Community-Centered Research*).

## 5. Conclusions

Successful community engagement can capitalize on a humanistic approach to research in the community by exploring individuals’ deep-seated values. This study proposes a process by which research can become community-centered where the values of participants are embodied in the design of the study and how the study is framed and conveyed. This then gives way to an opportunity to co-create the significance and meaning of the study with participants, making the initiative more likely to be successful in the long run. Researchers can benefit by connecting fields, such as public health, psychology, multimedia, design and the arts, among others, to meet the challenges of community-centered initiatives [7]. It is important to remember that community engagement often starts from a conversation and a simple question: “Who is this *person* that I am talking to?” Presence and respect for participants often move the conversation forward and helps research participants to feel heard, seen, and understood. Once this mindset is established, individuals are no longer just research participants who provide data. Instead, they are active co-creators of their communities, having a voice in matters that matter to them. Additionally, as this paper has illustrated, in-depth qualitative approaches can then effectively uncover the deeper stories and values that are key to building a connection between them and a study.

## Figures and Tables

**Table 1:** Values derived from the qualitative interviews. Examples and definition.

Theme and Sub-Themes/Value and Example	Schwartz’s Definition [39]
1. Benevolence: The majority of participants considered the connection with and care for their loved ones as the driving motivational forces governing their lives. The Benevolence theme incorporates three sub-themes: (1) Family and friends as a source of emotional support, encouragement, and inspiration (2) Intergenerational learning: learning from each other to help one another (3) Golden Rule: treat others the way you want to be treated (e.g., “*My family is the most important to me (…) even though sometimes life gets hard, especially here in New York, I’m just trying to live day by day and still be happy and enjoy life (…) it is important to have that support system.*”)	“Benevolence derives from the basic requirement for smooth group functioning and from the organismic need for affiliation. Most critical are relations within the family and other primary groups. Benevolence values emphasize voluntary concern for others’ welfare (helpful, honest, forgiving, responsible, loyal, true friendship, mature love, sense of belonging, meaning in life, a spiritual life).”
2. Universalism: It was critical for the majority of participants to make a difference in their communities by being involved in a cause or activity that improved the welfare of others, animals as well as nature. The Universalism theme incorporates three sub-themes: (1) Nature and a clean environment (2) Making a positive contribution in the world (3) Societal concern and appreciation of diversity (e.g., equality, social justice) and the complexity of human nature (e.g., “*Activism, social justice issues are very important to me (…) I believe that minority groups slowly energize and mobilize themselves to bring about change, but this change happens slowly, just like Martin Luther King said “the wheels of justice, they turn slowly, but they make a difference.*”)	“Defining goal: understanding, appreciation, tolerance, and protection for the welfare of all people and nature. Universalism derives from the survival needs of individuals and groups. Universalism combines two subtypes of concern—for the welfare of those in the larger society and world and for nature (broadmindedness, social justice, equality, world at peace, the world of beauty, unity with nature, wisdom, protecting the environment, inner harmony, a spiritual life).”
3. Self-direction: The majority of participants expressed a strong desire to grow and learn continuously in different areas in their lives, to pursue their interests and hobbies, to create new experiences, and to freely explore new goals. The Self-direction theme incorporates four sub-themes: (1) Self-directed learning as self-improvement, and problem-solving tool (2) Learning as a self-discovery: creating one’s own unique path (3) Self-direction as a pursuit of one’s hobbies and passions (4) Finding one’s tribe and seeking inspiration (e.g., “*I love learning from other people. This world is so colorful. There is so much to be learned.*”)	“Defining goal: independent thought and action-choosing, creating, exploring. Self-direction derives from organismic needs for control and mastery and interactional requirements of autonomy and independence (creativity, freedom, choosing own goals, curious, independent, self-respect, intelligent, privacy).”

## Data Availability

The data are not publicly available due to confidentiality protection, and ethical obligations of ensuring the security and protection of participants involved in qualitative research.

## Appendix A

Questions used in the psychological interview probing participants’ deep-seated life-motivating values:

1. Tell me a bit about yourself. What is your cultural background? (Probe 1: We all have different identities and roles related to our professional and personal life through which we express who we are. How would you define yourself? Probe 2: Think of different identities, such as ethnic, racial, but also social (e.g., mother, son, professional etc.) identities. Which of those identities are the most important to you?)
2. Where did you grow up?
3. What is important to you in your life? (Probe: What do you value?)
4. What is essential and critical to you in your life (Probe: Think of something that you cannot survive without or that would be very hard to live without)
5. What inspires you—what keeps you going? Do you have a life mantra, a life philosophy that you live by?
6. Who inspires you? Who is the most significant role model in your life? What about the person that makes him or her special?
7. What is the best advice that you have ever gotten? What is the best advice that you have ever given?
8. What makes your daily life enjoyable?
9. What makes your everyday life challenging? What helps you deal with these challenges?
10. What is it that you want, but you don’t have? What would help you get there?
11. What is it that you like doing?
12. Imagine that life is one big classroom, what is it that you would like to learn or know about that you believe would help you lead a more fulfilling life? (Probe: What is the one question that you wonder about?)
13. Is there anything else you would like to add or share with me?

Thank you very much for taking the time to chat with me today.
